# Anti-Inflammatory, Immunomodulatory, and Heme Oxygenase-1 Inhibitory Activities of Ravan Napas, a Formulation of Uighur Traditional Medicine, in a Rat Model of Allergic Asthma

**DOI:** 10.1155/2011/725926

**Published:** 2010-10-05

**Authors:** Sajida Abdureyim, Nurmuhammat Amat, Anwar Umar, Halmurat Upur, Benedicte Berke, Nicholas Moore

**Affiliations:** ^1^First Affiliated Hospital, Xinjiang Medical University, Xinjiang 830011, China; ^2^Department of Traditional Uighur Medicine, Xinjiang Medical University, Xinjiang 830011, China; ^3^Department of Pharmacology, Université de Bordeaux, 33076 Bordeaux, France

## Abstract

*Ravan Napas* (RN) is a traditional formula used to treat pulmonary symptoms and diseases such as coughing, breathing difficulty, and asthma in traditional Uighur medicine. The purpose of this study was to investigate the anti-inflammatory, and immuno-modulatory activity of RN in a well-characterized animal model of allergic asthma. Rats were sensitized with intraperitoneal (ip) ovalbumin (OVA) and alum, and then challenged with OVA aerosols. The asthma model rats were treated with RN; saline- and dexamethasone- (DXM-) treated rats served as normal and model controls. The bronchoalveolar lavage fluid (BALF) cellular differential and the concentrations of sICAM-1, IL-4, IL-5, TNF-*α*, INF-*γ*, and IgE in serum were measured. Lung sections underwent histological analysis. The immunohistochemistry S-P method was used to measure the expression of ICAM-1 and HO-1 in the lung. RN significantly reduced the number of inflammatory cells in BALF and lung tissues, decreased sICAM-1, IL-4, IL-5, TNF-*α*, and IgE in serum, and increased serum INF-*γ*. There was a marked suppression of ICAM-1 and HO-1 expression in the lung. Our results suggest that RN may have an anti-inflammatory and immuneregulatory effect on allergic bronchial asthma by modulating the balance between Th1/Th2 cytokines.

## 1. Introduction

Asthma is a major public health problem worldwide. Asthma morbidity and mortality have increased in the recent decades [1]. Bronchial asthma is a chronic inflammatory disorder of the airways characterized by airflow obstruction, airway inflammation, persistent airway hyperresponsiveness (AHR), and airway remodeling [2]. Increasing evidence suggests that many cells and cellular elements play prominent roles in the pathogenesis of allergic asthma [3, 4]. This pathogenesis is mediated by nonspecific infiltration by various inflammatory cells such as eosinophils, T-lymphocytes, macrophages, neutrophils, and epithelial cells [5, 6]. In addition, symptoms are mediated by a wide range of compounds such as histamine, cytokines, and cyclo-oxygenase and lipoxygenase products [7].

A variety of cytokine-bronchial cell interactions play an important role in normal host defense as well as in the pathogenesis of inflammatory airway disorders such as asthma, acute and chronic bronchitis, and bronchiectasis [8]. Increasing clinical and experimental evidence suggests that an imbalance between Th1 and Th2 leads to the clinical expression of allergic disease including asthma. The Th2 cytokines such as IL-4, IL-5, and TNF-*α* become overly abundant when activated by CD_4_^+^ T cells relative to Th1 cytokine (IFN-*γ*), and this is seen to play a central role in the pathogenesis of allergic asthma [9–13]. Antigen-induced IgE production, airway inflammation, and airway hyperresponsiveness have been well documented in patients with allergic asthma and in animal models [14–18].

 Adhesion of inflammatory cells to the bronchial epithelium is the crucial step in inducing bronchial inflammation in asthma. ICAM-1 participates by inducing the adhesion to and migration of the inflammatory cells through the endothelium [19]. Upregulation of ICAM-1 in epithelial and endothelial cells is believed to be a hallmark of asthma in adults [20, 21]. ICAM-1 is shed by the cell and can be detected in plasma, serum, or bronchoalveolar lavage fluid (BALF) in a soluble form, sICAM-1. The role of heme oxygenase (HO) in pulmonary medicine is a rapidly emerging field [22–24]. HO-1 can be induced by various stimuli such as stress, endotoxins, hypoxia, and so forth. The highly active synthesis of HO-1 proteins has been found in bronchial epithelial cells, lung tissues, lung macrophages and type II lung epithelial cells of asthma models [25, 26]. Expression of the HO-1 is increased within the lung tissue in allergic airway inflammation, and overexpression of HO-1 could enhance allergic airway inflammation [27, 28].

 The treatment of asthma has been improved by the implementation of management guidelines in recent years, with further development in the studies that identify the mechanisms of asthma. Inhaled corticosteroids and *β*-2-agonists are used as the first line of treatment of asthma, reducing airway inflammation and bronchial constriction effectively. However, the effects of these drugs are not always satisfactory in clinical practice because of local or systemic side effects [29]. Therefore, there is a place for new or alternative approaches to the control of asthma such as those found in complementary and alternative medicine (CAM), and especially in traditional medical systems. With the development of modern pharmacological and molecular biology techniques, studying the mechanisms of CAM in treating asthmatic diseases is increasingly important and potentially useful.

 CAM approaches include those such as traditional Uighur medicine (TUM), which is used in Xinjiang Uighur Autonomous Region of China. From the principle of TUM, the pathogenesis of asthma is due to the stimulation of Abnormal Hilit (the fluids involved as the basis of traditional Uighur medicine) in the lung tissue, which weakens natural power factors and induces inflammation. *Ravan Napas* (RN) is a TUM formula, which has been used for more than 2000 years [30]. RN is a herbal concoction, which has been used under TUM principles with the ultimate goal of relief from wheezing and reduction of frequency of attacks in asthmatic patients. Physicians in the TUM system use RN that has been manufactured into granules to remove the Abnormal Hilit and to boost the body's natural power. RN is regarded as a potent tonic to increase energy levels and stimulate the immune system. Several studies have focused on its immune-regulating properties, and RN has been shown to increase human lymphocyte proliferation, and immunoglobulin production in normal mice [31]. Plants related to those used in RN [32, 33] and other plants used in traditional Asiatic medicines [34–40] have been shown to have an effect on immunomodulation and inflammatory responses in experimental asthma. However, RN has not yet been investigated for application to allergic diseases. We used the ovalbumin- (OVA-) induced asthmatic rat model to evaluate possible mechanisms of RN on inflammation and on systemic immune responses, using OVA-induced BALF cell proliferation, cytokines production, and expression of ICAM-1 and HO-1 in the lung.

## 2. Methods

### 2.1. RN Preparation

The composition of RN is listed in Table 1 and the chemicals identified in these herbs are listed in Table 2. The constituent plants were purchased from Xinjiang Autonomous Region Traditional Uighur Medicine Hospital (Urumqi, China) and were authenticated by associate chief pharmacist Anwar Talip. In accordance with the protocol of preparation, 1 kg dried herb powder was soaked in 10 L of warm distilled water for 12 hours and boiled for 1 hour. The extract was filtered and concentrated under reduced pressure and low temperature (60°C) on a rotary evaporator, dried in vacuum conditions, and stored in the refrigerator. The yield of the extract was found to be 22.1%.

### 2.2. Animals and OVA-Induced Asthmatic Rat Model

Male Wistar Rats, weighing 200–250 g were purchased from Xinjiang Medical University Animal Centre. The animals were housed in stainless steel cages in the Good Laboratory Practices-compliant laboratory of the Xinjiang Traditional Uighur Medicine Institute. The room temperature (22 ± 1°C) and humidity (55 ± 10%) were controlled automatically. The experimental procedures were approved by the guidelines of the Animal Care and Use Committee of Traditional Uighur Medicine Institute, Xinjiang Uighur Autonomous Region. Induction of asthma in rats has been described previously [41]. Rats were immunized with an intraperitoneal injection of a suspension containing 40 mg of OVA (Sigma Chemical Co., St. Louis, MO, USA) and 2 mg of aluminium hydroxide (Beijing Chemical Reagent Factory). Fifteen days after immunization, rats were challenged by exposure to a 1% OVA in phosphate-buffered saline (PBS) aerosol once daily during 20 min per day for 8 consecutive days. The challenge was carried out in a half vented metal chamber (35 cm × 25 cm × 15 cm) by using an ultrasonic nebulizer, Aerosol flow rate was 3 mL/min. Control group rats were exposed to nebulised sterile saline using the same method. One day after the last challenge the blood and tissue sample were collected.

### 2.3. Experimental Groups and Treatments

**Figure 1:**
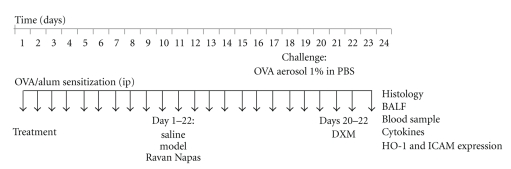
*Experimental protocol.* Rats were immunized with intraperitoneal injection of a suspension containing 40 mg ovalbumin (OVA) and 2 mg aluminium hydroxide. 15 days after the immunization, rats were challenged by exposure to an aerosol of 1% OVA in PBS for 20 minutes once daily for 8 consecutive days (days 15 to 22). Three groups were treated for 22 days after sensitization, with saline (controls), RN1, and RN2 (RN orally at 0.25 g/kg or 0.5 g/kg per day). DXM group was treated with dexamethasone 10 mg/kg on days 20, 21, and 22.

The rats were divided into five groups (ten rats per group): normal, OVA-control, OVA-RN1 group, OVA-RN2 group, and Ova–DXM group. All OVA groups were sensitized and challenged with OVA as described above. (Figure 1) OVA-control rats were administered saline orally for 22 days after first sensitization. OVA-RN1 and OVA-RN2 were two RN dosage treatment groups, administered with RN orally for 22 days after first sensitization at the dose of 0.25 g/kg (RN1) and 0.5 g/kg (RN2) per day, respectively. Ova–DXM rats were administered Dexamethasone (DXM) on days 20, 21, and 22, before challenge, at the dose of 10 mg/kg.

### 2.4. Measurement of Serum sICAM-1, IL-4, IL-5, TNF-*α*, INF-*γ*, and Ige

Rats were anesthetized with an intraperitoneal injection of sodium pentobarbitone (100 *μ*g/kg), blood was collected and centrifuged at 4°C (3000 rpm) for 10 min, and the serum was stored at −80°C for measurement of cytokines and IgE. ELISA kits from R&D Systems were employed for the measurement of sICAM-1, IL-4, IL-5, TNF-*α*, INF-*γ*, and IgE.

### 2.5. BALF Preparation and Cell Differential Counts

After blood collection, the trachea was cannulated and the right bronchi were tied for histological studies. Bronchoalveolar lavage fluid (BALF) was collected by lavaging the left lung via the trachea with 15 mL of ice-cold PBS. After five lavages, approximately 10 mL of BALF were recovered and centrifuged at 4°C (1500 rpm) for 10 minutes. The cells in the BALF were resuspended in 100 *μ*L of PBS for total and relative leukocyte counts using a hemocytometer. The cells in the BALF were resuspended in normal saline after a brief hypotonic exposure to lyse red blood cells, then immediately placed on the hemocytometer, left unmoved for 3–5 minutes, and then counted in 10-square chambers.

### 2.6. Histological Analysis

After sacrifice, noninflated lungs were removed, fixed with 10% buffered formalin, and processed in a standard manner. Tissue sections were stained with hematoxylin-eosin and examined microscopically. Peribronchial cells were counted using a five-point scoring system to estimate the severity of leukocyte infiltration. The leukocyte scoring was examined in three independent fields of lung section from each rat. Mean scores were obtained from ten rats. The scoring system was 0, no cells; 1, a few cells; 2, a ring of cells 1 cell layer deep; 3, a ring of cells 2–4 cell layers deep; and 4, a ring of cells more than 4 cell layers deep.

### 2.7. Immunohistochemistry Analysis

To identify the source of HO-1 and ICAM-1 positive cells, immunohistochemistry staining was performed. Antibodies were diluted 1 : 200 with 1% normal goat serum (NGS) in PBS. Lung sections were sequentially incubated overnight at 4°C in the dark with rabbit anti-HO-1 or sheep anti-ICAM-1, followed by biotin-labelled antibody to rabbit IgG or sheep IgG and incubated for 30 min in streptavidin-peroxide, coloured with 3,3-diaminobenzidine (DAB) then sealed with neutral gum. The expression of ICAM-1 and HO-1 was located in the cytoplasm. Brown-yellow granules were considered as positive expression. The scoring system was the number of the positive staining cells: 0, no cells; 1, 0 ~ 10 cells; 2, 11 ~ 24 cells; 3, 25 ~ 49; 4, 50 ~ 74 cells; and 5, >75 cells. All histology and immunohistochemical analyses were done blind to treatment group.

### 2.8. Statistical Analysis

The statistical significance of any difference was determined by one-way ANOVA followed by Tukey's protected *t*-tests when ANOVA was significant. The data are expressed as mean ± S.E.M. The SPSS statistical software package (Version 10.0, Chicago, IL) was used for the statistical analysis. 

## 3. Results

### 3.1. Total Leukocytes and Eosinophils in BALF

**Figure 2:**
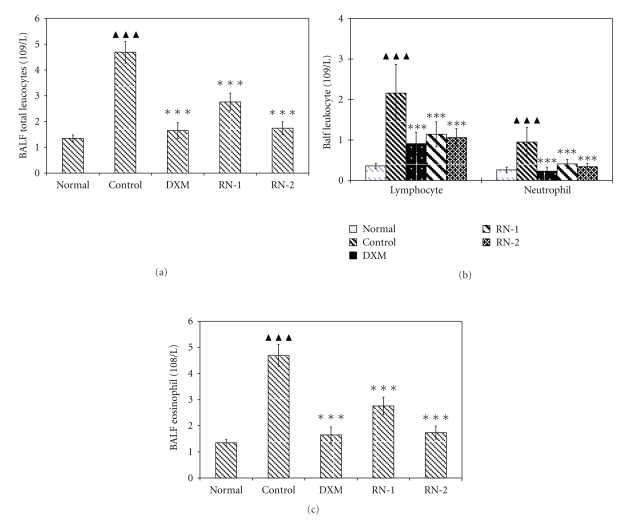
*Effect of Ravan Napas (RN) on the recruitment of inflammatory cells in BALF obtained from OVA-induced rat model of asthma.* Rat Bronchoalveolar Lavage Fluid (BALF) was harvested during the 24 h after last OVA challenge. All rats were sensitized with OVA: (a) total leucocytes, (b) lymphocytes and neutrophils, and (c) eosinophils. Normal: rats sensitized with OVA and challenged with saline. Control: rats sensitized and challenged with OVA. DXM: OVA-sensitized and challenged rats treated with Dexamethasone (10 mg/kg). RN-1: OVA-sensitized and challenged rats treated with RN (0.25 g/kg/day). RN-2: OVA-sensitized and challenged rats treated with RN (0.5 g/kg/day). Data are expressed as mean ± S.E.M., *n* = 10 rats per treatment group. ^▲▲▲^ *P* < .01 versus Normal; ***P < .01 versus Control.

RN significantly decreased the number of leukocytes in BALF in comparison with the control group (Figures 2(a) and 2(b) *P* < .01 versus normal). Differential leukocyte counts showed that RN decreased the number of neutrophils, eosinophils, and lymphocytes in comparison with the control group (*P* < .01) (Figure 2(c)).

### 3.2. Pathological Inflammation in Lung Tissue

**Figure 3:**
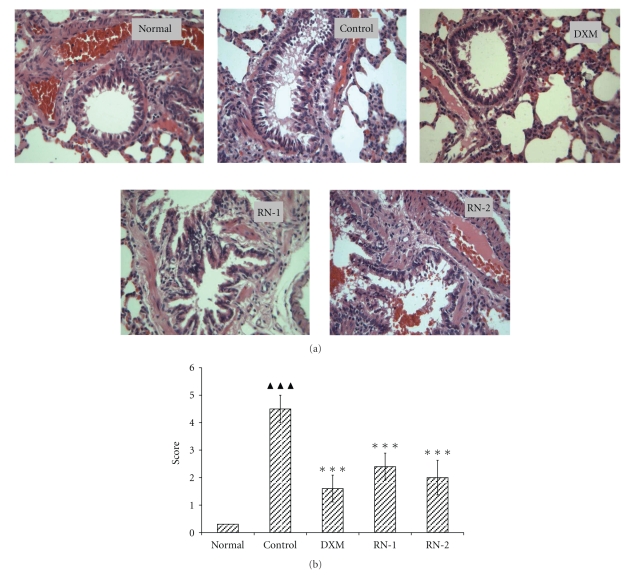
*Effect of Ravan Napas (RN) on pulmonary inflammation in OVA-induced rat model of asthma.* Lung tissues were obtained on the day after the last OVA challenge. Tissues were stained with hematoxylin and eosin (H&E, 400x) (a). The inflammatory cell infiltration in the lung tissues was scored as described in the method section (b). Normal: rats sensitized with OVA and challenged with saline. Control: rats sensitized and challenged with OVA. DXM: OVA-sensitized and challenged rats treated with Dexamethasone (10 mg/kg). RN-1: OVA-sensitized and challenged rats treated with RN (0.25 g/kg/day). RN-2: OVA-sensitized and challenged rats treated with RN (0.5 g/kg/day). Data are expressed as mean ± S.E.M., *n* = 10 rats per treatment group. ^▲▲▲^ *P* < .01 versus Normal; ***P < .01 versus Control.

Rats from each group were autopsied, and sections of the major organs were examined by a pathologist unaware of their origin. No gross or histological abnormalities were observed in tissues other than the lung. As previously described, lungs from normal (sham-treated)/antigen-sensitized/challenged mice contained large numbers of peribronchial and perivascular eosinophils (data not shown). The lung tissue obtained from ovalbumin-induced asthmatic rats was characterized by dense peribronchial inflammation due to leukocyte infiltration and mucus hyperproduction by goblet cells within the bronchi when compared with normal tissue. This inflammation resulted in the narrowing of the bronchi (Figure 3(a)). RN extract significantly reduced the degree of inflammatory cell infiltration (*P* < .05) (Figure 3(b)). 

### 3.3. Serum sICAM-1, IgE, IL-4, IL-5, TNF-*α*, and INF-*γ*

**Figure 4:**
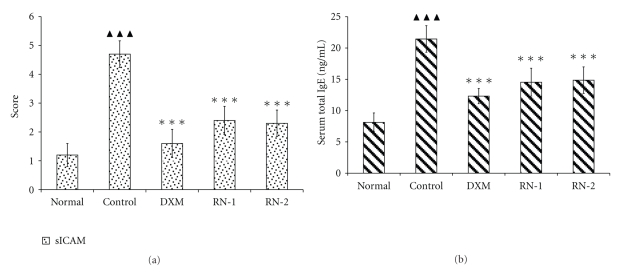
*Effect of Ravan Napas (RN) on sICAM-1 and total IgE concentration in serum of OVA-induced rat model of asthma*. Blood was sampled and serum obtained 24 h after last OVA challenge. sICAM-1 and total IgE were measured by ELISA as described in the materials and methods section. (a): sICAM-1 concentrations; (b): IgE concentrations; Normal: rats sensitized with OVA and challenged with saline. Control: rats sensitized and challenged with OVA. DXM: OVA-sensitized and challenged rats treated with Dexamethasone (10 mg/kg). RN-1: OVA-sensitized and challenged rats treated with RN (0.25 g/kg/day). RN-2: OVA-sensitized and challenged rats treated with RN (0.5 g/kg/day). Data are expressed as mean ± S.E.M., *n* = 10 rats per group.  ^▲▲▲^ *P* < .01 versus Normal; ***P < .01 versus Control.

**Figure 5:**
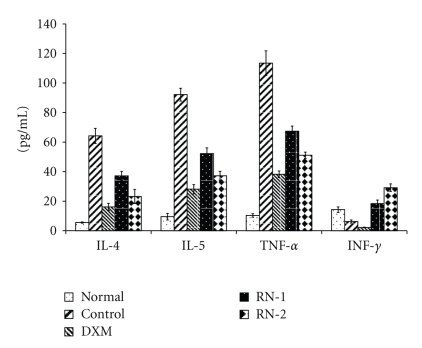
*Effect of Ravan Napas (RN) on IL-4, IL-5, INF-*α*, and INF-*γ* in serum of OVA-induced rat model of asthma.* Blood was sampled and serum obtained 24 h after last OVA challenge. IL-4, IL-5, TNF-*α*, and INF-*γ* were analyzed by ELISA as described in the materials and methods section. Normal: rats sensitized with OVA and challenged with saline. Control: rats sensitized and challenged with OVA. DXM: OVA-sensitized and challenged rats treated with Dexamethasone (10 mg/kg). RN-1: OVA-sensitized and challenged rats treated with RN (0.25 g/kg/day). RN-2: OVA-sensitized and challenged rats treated with RN (0.5 g/kg/day). Data are expressed as mean ± S.E.M., *n* = 10 rats per group. ^▲▲▲^ *P* < .01 versus Normal; ***P < .01 versus Control.

The serum concentration of sICAM-1 and total IgE in model group significantly increased following the induction of asthma (*P* < .01) (Figure 4). OVA induced IL-4, IL-5, TNF-*α*, and IgE elevations in serum (Figure 5). RN inhibited sICAM-1, and IgE increases in serum (*P* < .05) (Figure 4). IL-4, IL-5, and TNF-*α* levels in serum were reduced in RN-treated rats compared with model group rats (Figure 5).

The INF-*γ* concentration was reduced by OVA exposure and increased significantly in RN-treated rats. INF-*γ* concentrations after RN1 and RN2 were increased significantly compared with control and normal controls (Figure 5).

### 3.4. Expression of ICAM-1 and HO-1 in Lung

**Figure 6:**
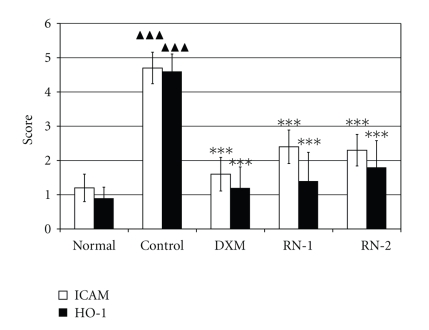
*Effect of RN on the expression of ICAM-1 and HO-1 in lung tissue of OVA-induced rat model of asthma*. Wistar rat lung tissue was obtained 24 hours after last Ova challenge. Immunohistochemistry was performed as described in materials and methods section. Expression of ICAM-1 and HO-1 in the lung tissues was scored as described in Section 2. Normal: rats sensitized with OVA and challenged with saline. Control: rats sensitized and challenged with OVA. DXM: OVA-sensitized and challenged rats treated with Dexamethasone (10 mg/kg). RN-1: OVA-sensitized and challenged rats treated with RN (0.25 g/kg/day). RN-2: OVA-sensitized and challenged rats treated with RN (0.5 g/kg/day). Data are expressed as mean ± S.E.M., *n* = 10 rats per group. ^▲▲▲^ *P* < .01 versus Normal; ***P < .01 versus Control.

RN strongly inhibited the increased expression of ICAM-1 and HO-1 following the induction of asthma (*P* < .05) (Figure 6) when compared with model control rats.

## 4. Discussion

It has been reported that some antiasthma CAM formulas have therapeutic effects on allergic asthma [73–76]. Inhibition of the regional inflammatory response through the reduction of antigen-induced inflammatory cells and inflammatory cytokines production is a main therapeutic objective in the treatment of asthma. In this study, a rat model of allergic asthma was developed, the lung tissues examined, and BAL analysed to assess the effect of RN on airway inflammation in experimental asthma. Pathology analysis documented that 22-day RN administration reduced the allergic inflammatory infiltration in the lung tissues, reduced OVA-induced sICAM-1, IL-4, IL-5, TNF-*α*, and IgE elevations in serum, and increased INF-*γ* concentration. We also found that RN markedly suppressed increased ICAM-1 and HO-1 expression in lung tissue of allergic asthma rats. Collectively, all of these results demonstrated that RN is a potent agent in inflammatory pulmonary diseases.

The precise mechanisms of chronic airway inflammation in asthma are incompletely known but are considered to be dependent on the sustained infiltration and activation of many inflammatory cells including lymphocytes, eosinophils, basophils, and macrophages, followed by synthesis and release of a variety of proinflammatory mediators and cytokines [77, 78]. Th2 lymphocytes are the key orchestrators of this inflammation, initiating and propagating inflammation through the release of their cytokines, IL-4, IL-5, and TNF-*α* in turn recruiting and activating eosinophils, the effector cells in asthma [79–82]. The infiltration of eosinophils into the airways has been linked to the production of IL-5, which is important for eosinophil proliferation, activation, and migration [83]. IL-4 induces IgE isotype switching in B lymphocytes [84] and mucus production by goblet cells [85], as well as upregulation of the expression of adhesion molecules required for inflammatory cell recruitment [79]. TNF-*α* is also an important chemoattractant for the recruitment of eosinophils into the lungs [10, 86]. It is also a potent modulator of immune and inflammatory response.

Migration of leukocytes from circulation into tissue is dependent upon the interaction with adhesion molecules expressed on the cell surface and endothelium. ICAM-1 and its soluble form, sICAM-1, play important roles in the development of airway/lung inflammation in asthma. When inflammation occurs, the expression of ICAM-1 on the bronchial epithelium and lung vascular endothelium is significantly increased and thereby increases the adhesion of eosinophils on the epithelium [87–89]. There is a close correlation between high concentration of sICAM-1 and high expression of ICAM-1 with the severity of asthma in patients and asthmatic rats [19, 89, 90]. Higher concentrations of sICAM-1 in serum and BALF reflect the upregulation of ICAM-1 expression in allergic bronchial asthma, and these high concentrations may contribute to the pathogenesis of atopic bronchial asthma [91, 92].

The eosinophil is regarded as a key mediator of the pathology and abnormal physiology of bronchial asthma [93]. It has been suggested that eosinophils contribute to tissue damage [94, 95] and airway inflammation. AHR also may play an important role in recruitment of T cells to the lung during airway allergic responses by modulating chemokine and cytokine production in the lung. In most asthma phenotypes, there are increases in eosinophils in the tissues, blood and bone marrow and, in general, raised numbers correlating with disease severity [96]. Specifically, TNF-*α* is an important chemoattractant for the recruitment of eosinophils into the lungs [10, 86]. It is also a potent modulator of immune and inflammatory response. IL-5 uniquely and specifically participates in the control of eosinophil production and differentiation [10]. Attenuated synthesis of TNF-*α* and IL-5 could therefore relieve allergic responses caused by eosinophils.

**Figure 7:**
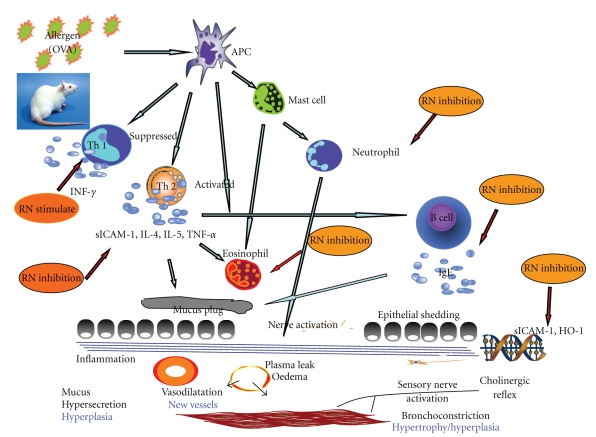
*Main anti-inflammatory and immunoregulatory targets of Ravan Napas (RN) in allergic asthma.* This figure illustrates the cascade of activations and inactivations resulting from exposure to allergen after sensitization in asthma, from exposure of Antigen-presenting cells (APC) to activation of mast cells and neutrophils, activation of Th2 cells liberating ICAM, interleukins, and TNF*α*, inactivation of Th1 cells releasing INF*γ*, and so forth. From the results reported, RN, as indicated, acts on various pathways implicated in the bronchial response to antigens: stimulation of Th1, inactivation of Th2, decreased ICAM and HO-1 expression, decreased IgE production, and decreased recruitment of neutrophils and other inflammatory cells.

Our results showed that serum concentrations of IL-4, IL-5, TNF-*α*, and sICAM-1 were significantly reduced in allergic rats after RN administration. In addition, RN inhibited the pulmonary accumulation of leukocytes and eosinophils in rats, which is parallel to the decrease of IL-4, IL-5, TNF-*α*, and sICAM-1 in serum and overexpression of ICAM-1 and HO-1 in allergic lung tissue (Figure 7). Our study is similar to previous reports and consistent with reports of increased ICAM-1 expression in the lungs of patients with asthma [97]. Cell count analyses and histological results corroborate the positive correlations of the inflammatory cells of BALF with the extent of total inflammatory cells, eosinophil, lymphocyte, and macrophage infiltration in lung tissues and BALF. In this study, the inhibitory effect of RN on inflammation in allergic rats was accompanied by a significant decrease in Th2 cytokines, sICAM-1, and ICAM-1 overexpression. Our results suggest that RN may play a key role in blocking the recruitment of leukocytes and eosinophils in lung through Th2-cytokines dependent pathway, much as dexamethasone. 

As expected, DXM, one of the most potent corticosteroids, also suppressed antigen-induced AHR and eosinophilic inflammation in this model. However unlike RN, DXM suppressed Th1 responses (IFN-*γ*) as well as Th2 (IL-4, IL-5, and TNF-*α*) responses. In this study, the administration of RN resulted in a downregulation of the Th2 cytokines IL-4, IL-5, and TNF-*α*; however, there was significant concomitant increase in the levels of the Th1 cytokine IFN-*γ* in serum. In addition, IFN-*γ* levels were higher in the RN group than those in the model group or Normal group. This type of immunoregulation may prove to be more beneficial than Th1 cytokines (IFN-*γ* and IL-12) or Th1 adjuvant therapy, which may cause undesirable inflammation because of higher-than-normal levels of Th1 cytokines. These findings suggest that RN and perhaps other antiasthma formulas may offer some clinical advantages over corticosteroids because they are less likely to increase the patient's susceptibility to infection.

Oxidative stress is thought to induce the inflammation of many chronic diseases [98]. During asthma, reactive oxygen species (ROS)—including superoxide anion—is produced in tissues, from oxidative injury [24]. One of these defence mechanisms is the induction of a stress-response protein, HO-1. HO-1 catalyses the initial and rate limiting step of heme metabolism. In asthma attacks, since the activity and protein synthesis of HO-1 are greatly increased, its products, bilirubin, free iron, and CO would also be increased [99, 100]. CO can act as second messenger [101]. Although studies suggest a cytoprotective effect of HO-1, its overexpression might also contribute to asthma inflammation. The following mechanism might be involved: HO-1 and CO accelerate the release of proinflammatory mediators such as IL-5 and TNF-*α* in mast cells and eosinophils thus increasing the hyperresponsiveness of the airways [102]. Bilirubin, if produced excessively, would disturb the biomembrane. Free iron, if it accumulates, could become cytotoxic. So we can assume that during asthma, HO-1 has dual effects [103]. We also found that low levels of HO-1 expression are protective. Moderate levels of expression decrease its cytoprotective effect, whereas high expression actually worsens the damage. The experimental results showed that the expression of HO-1 was significantly higher in the model than in the control group. Interestingly, similar results have been reported with extracts from plats close to those used in RN [32, 33].

In conclusion, RN suppressed antigen-induced inflammation in this rat model of asthma. This effect was accompanied by reduction of leukocytes, eosinophilic inflammation, and specific downregulation of Th2 responses, inhibiting the expression of ICAM-1 and HO-1 in lung tissue. This study suggests that Uighur herbal medicines, such as RN, should be further explored for possible use for treatment of asthma and allergy.

## Figures and Tables

**Table 1:** Plants contained in Uighur herbal formula: Ravan Napas.

Latin name	Family	Part used	Vulgar name	Uighur name
*Hyssopus cuspidatus* Boriss.	Lamiaceae	Aerial part	Hyssop	Zupa
*Foeniculum vulgare* Mill.	Apiaceae	Root	Fennel	Arpa Badian
*Carthamus tinctorius* L	Asteraceae	Seed	Safflower	Zarangza Uruki
*Brassica rapa* L	Brassicaceae	Seed	Turnip mustard	Qamgur Uruki
*Malva verticillata* L.	Malvaceae	Seed	Mallow	Binapxa Uruki
*Astragalus mongholicus *Bunge	Fabaceae	Root	Vetch	Katira
*Ziziphus jujuba *Mill	Rhamnaceae	Fruit	Red date	Qilan
*Viola tianshanica *Maxim	Violaceae	Aerial part	Tienshan Violet	Gul binapxa

**Table 2:** Chemical composition of the plants contained in Uighur herbal formula: Ravan Napas.

Plant	Major chemical components	References
*H. cuspidatus* aerial part	Essential oil: germacrenes B and D, hexadecanoic acid, (+)-transcaryophyllene, (+)-spathulenol	Ablizl 2009 [42]

*F. vulgare *fruit	transanethole, p-anisaldehyde, chlorogenic acid, and quercetin-3-O-*β*-D-glucuronide	Bilia 2000 [43] Kitajima 1998 [44–46] Ishikawa 1998 [47] Ishikawa 1999 [48] Kitajima 1999 [49] Ono 1995 [50] Ono 1996 [51] De Marino 2007 [52]
	*Water-soluble constituents: *	
	alkyl glycosides, p-hydroxyphenylpropylene glycol glycosides, phenylpropanoid glycosides, benzyl alcohol derivative glycosides, phenylethanoid and its glycoside, threo-epoxyanethole glycoside, fenchane-, norfenchane-type monoterpenoid glycosides, menthane-, thujane-, camphane-type monoterpenoids, and monoterpenoid alcohols.	
	Monoterpene glycosides: *β*-D-glucopyranosides of (1S,2R,4S)-2,4-dihydroxy-1,8-cineole-2-O, (1R,4R,6R)-4,6-dihydroxy-1,8-cineole-6-O, (1R,4R,6R)-4,6-dihydroxy-1,8-cineole-4-O, (1S,2R,4R,6S)-2,6-dihydroxy-1,8-cineole-2-O and (1S,2R,4S,5R)-2,5-dihydroxy-1,8-cineole-2-O	
	Zizybeoside I, icaviside A4, syringin, sinapyl alcohol 1,3′-di-O-*β*-D-glucopyranoside, threo-anethole glycol, and erythro-anethole glycol glycosides.	
	Stilbene trimers: 11a-O-*β*-D-glucopyranoside, 13b-O-*β*-D-glucopyranoside, 11a,13b-di-O-*β*-D-glucopyranoside, 11a,13b,13c-tri-O-*β*-D-glucopyranoside of cis-miyabenol C	
	Cis-miyabenol C 11a-*O*-*β*-d-glucopyranosyl-(1→6)-*β*-d-glucopyranoside, *cis*-miyabenol C 11a,13c-di-*O*-*β*-d-glucopyranoside.	

*C. tinctorius *seed	Serotonin derivatives, lignans, and flavonoids.	Koyama 2006 [53] Kim 2007 [54] Ahmed 2000 [55]
	Phenolic compounds: matairesinol 4′-o-*β*-D-glucoside, 8′-hydroxyarctigenin 4′-0-*β*-D-glucoside, matairesinol, 8′-hydroxyarctigenin, N-feruloylserotonin 5-O-*β*-D-glucoside, N-(p-coumaroyl)-serotonin-5-O-*β*-D-glucoside, N-feruloylserotonin, N-(p-coumaroyl)serotonin, luteolin 7-O-*β*-D-glucoside, luteolin, acacetin 7-O-*β*-glucuronide, and acacetin	
	Acacetin 7-O-*β*-D-apiofuranosyl-(1′ → 6′)-O-*β*-D-glucopyranoside together with previously isolated kaempferol 7-O-*β*-D-glucopyranoside, acacetin 7-O-*α*-L-rhamnopyranoside, and acacetin.	

*B. rapa *seed	Isothiocyanates: 3-butenyl isothiocyanate.	Taveira 2009 [56]

*M. verticillata *seed	Polysaccharides: *β*-1,3- and *β*-1,6-linked D-galactosyl residues acidic polysaccharide (MVS-VI) and *β*-1,3-linked D-glucose, and D-galactose residues neutral polysaccharide (MVS-I).	Tomoda 1992 [57] Shimizu 1991 [58]

*A. mongholicus *root	Triterpene saponins, isoflavonoids, and polysaccharides.	Zhang 2007 [59] Chu 2010 [60] Han 2007 [61] Bian 2006 [62] Yu 2005 [63] Wang 2008 [64] Subarnas 1991 [65] Shimizu 1991 [66] Yan 2005 [67]
	Methanolic extract: cyclolanostane-type saponins including 8 astragaloside malonates, and malonylastragaloside I.	
	Astragalosides I, II, IV, acetylastragaloside I, isoastragaloside I isoastragaloside II, astramembrannin II, afrormosin, calycosin, calycosin-7-O-*β*-D-glucoside, daucosterol formononetin, formononetin-7-O-*β*-D-glucoside, formononetin-7-O-*β*-D-glucoside-6′′-O-malonate, 2′,4′-dimethoxy-3′-hydroxyisoflavan-6-O-*β*-glucopyranoside, (6aR,11aR)9,10-dimethoxypterocarpan-3-O-*β*-D-glucoside, (6aR,11aR)3,9-dimethoxy-10-hydroxypterocarpan, (3R)8,2′-dihydroxy-7,4′-dimethoxyisoflavan, ononin, pinitol, *β*-sitosterol.	
	Calycosin-7-O-*β*-D-glucoside, (6aR,11aR)-3-hydroxy-9,10-dimethoxypterocarpan-3-O-*β*-D-glucoside, and 3,9-di-O-methylnissolin 4-hydroxy-5-hydroxymethyl-[1,3]dioxoian-2,6′-spirane-5′,6′,7′,8′-tetrahydro-indolizine-3′-carbaldehyde (HDTIC-1 and -2).	
	Isoflavanoids: 7-O-methylisomucronulatol, isomucronulatol 7,2′-di-O-glucoside, 5′-hydroxyisomucronulatol 2′,5′-di-O-glucoside, and (3R)-7,2′-dihydroxy-3′,4′-dimethoxyisoflavan-7-O-*β*-D-glucoside *α*-arabino-*β*-3,6-galactan-type acidic polysaccharide	
	Lectin	

*Z. jujube *fruit	*Water and ethanol extracts: *	
	Terpenoids: ceanothic acid, alphitolic acid, zizyberanal acid, zizyberanalic acid, zizyberanone, epiceanothic acid, ceanothenic acid, betulinic acid, oleanolic acid, ursolic acid, 2*α*-hydroxyursolic acid and zizyberenalic acid, (2S,3S,4R,8E)-2-[(2′R)-2′-hydroxy-tetracosanoyl]-8-octadecene-1,3,4-triol and its 1-O-*β*-D-glucopyranosyl derivate, maslinic acid, 3*β*,6*β*-stigmast-4-en-3,6-diol, *β*-sitosterol, daucosterol, tetracosanoic acid, and heptadecanoic acid.	Guo 2009 [68–70] Pawlowska 2009 [71]
	Flavonoids: kaempferol 3-O-robinobioside, kaempferol 3-O-rutinoside, quercetin 3-O-robinobioside, quercetin 3-O-rutinoside, quercetin 3-O-*β*-D-xylosyl-(1-2)-*α*-L-rhamnoside, quercetin 3-O-*α*-L-arabinosyl-(1-2)-*α*-L-rhamnoside, and rutin.	

*V. tianshanica *aerial part	Daucosterol and flavonoids: isorhamnetin 3-O-*β*-glucoside, kaempferol, kaempferol 7-O-*β*-D-glucopyranoside, kaempferol 3-O-*β*-D- glucopyranoside, and quercetin.	Yu 2009 [72]
